# Real-time surgical navigation for intraoperative lymph node localization during targeted robot-assisted pelvic and retroperitoneal lymph node dissection

**DOI:** 10.1007/s00464-026-13187-5

**Published:** 2026-07-27

**Authors:** Marijn A. J. Hiep, Wouter J. Heerink, Harald C. Groen, Lisanne P. J. Venix, Laura Aguilera Saiz, Enzo Kerkhof, Henk G. van der Poel, Pim J. van Leeuwen, Maaike W. van de Kamp, Quirijn R. J. G. Tummers, Theo J. M. Ruers

**Affiliations:** 1 Department of Surgical Oncology, The Netherlands Cancer Institute, Plesmanlaan 121, 1066 CX Amsterdam, The Netherlands; 2 Department of Urology, The Netherlands Cancer Institute, Plesmanlaan 121, 1066 CX Amsterdam, The Netherlands; 3 Faculty of Science and Technology (TNW), Nanobiophysics Group (NBP), University of Twente, 7500 AE Enschede, The Netherlands

**Keywords:** Augmented reality, Robot-assisted pelvic lymph node dissection, Robot-assisted retroperitoneal lymph node dissection, Surgical navigation, Ultrasound

## Abstract

**Background:**

Localization of suspected metastatic LNs during targeted robot-assisted lymphadenectomy is challenging due to anatomical variability and surrounding vital structures. Image-guided surgical navigation may improve intraoperative LN localization and completeness of oncologic dissection. The aim of this study is to evaluate the accuracy and usability of surgical navigation for localization of suspected metastatic lymph nodes (LNs) during robot-assisted pelvic and retroperitoneal LN dissection.

**Methods:**

Patients with one or more suspected metastatic LNs on preoperative imaging scheduled for robot-assisted pelvic or retroperitoneal LN dissection were prospectively included. A patient-specific three-dimensional (3D) model was automatically registered intraoperatively using electromagnetically tracked ultrasound of the bone surface. Real-time navigation was displayed via the da Vinci® TilePro™ as a 3D model or augmented reality overlay. A tracked pointer or instrument was used for LN localization. Accuracy was defined as the shortest distance between the surgeon-indicated LN and the corresponding 3D model. Usability was assessed using the system usability scale (SUS).

**Results:**

Twenty patients with 40 target LNs were included. One para-aortic LN was inaccessible in a patient with a BMI of 52 kg/m^2^. The remaining 39 LNs were successfully localized using navigation with a median accuracy of 0.8 mm (IQR 0.0–3.0). Navigation supported surgical decision-making and LN localization, with a SUS score of 70 (IQR 64–73). Registration time was 7 (5–10) min. No navigation-related complications occurred.

**Conclusions:**

Surgical navigation enabled highly accurate LN localization during robot-assisted pelvic and retroperitoneal LN dissection. The technique is safe, feasible, and supports targeted oncologic surgery and intraoperative decision-making. In the future, wireless tracking systems or simultaneous localization and mapping techniques could allow broader clinical implementation of the navigation technique.

**Graphical abstract:**

**Figure:**
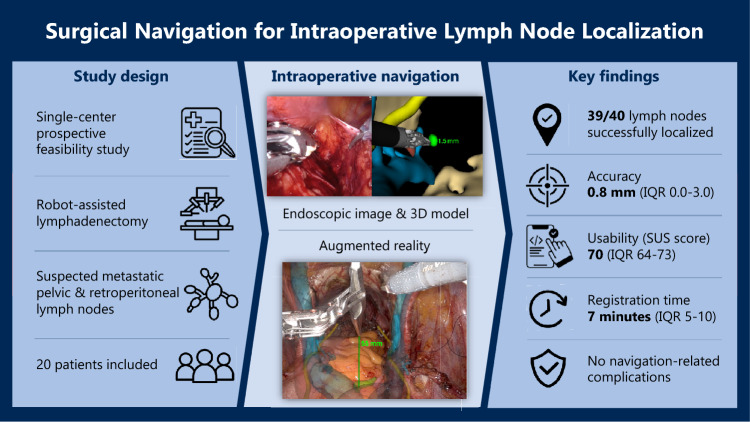

**Supplementary Information:**

The online version contains supplementary material available at 10.1007/s00464-026-13187-5.

Lymph node (LN) metastases play a significant role in prognosis and survival of cancer patients, and LN dissection is primarily performed for accurate staging and therapeutic management of the disease [1]. Surgical removal of metastatic LNs may reduce recurrence or further cancer spread, improving long-term survival [2]. For lower-abdominal nodal metastases, robot-assisted pelvic and retroperitoneal lymph node dissection (RA-PLND and RA-RPLND) have largely replaced open approaches due to lower morbidity and faster recovery [3, 4]. However, intraoperative identification and localization of target LNs remain challenging, especially for smaller or deeply located LNs [3, 5].

A novel technology to improve intraoperative localization of malignancies is image-guided surgical navigation. This technique combines preoperative imaging with tracked surgical instruments, enabling real-time feedback on their position relative to the patient’s anatomy. While this technology is already well established in orthopedic and neurosurgery with commercially available systems, e.g., Brainlab and Stryker [6, 7], pelvic soft-tissue navigation systems are still in development [8–13]. In a randomized controlled trial for open targeted LN dissection, the use of navigation resulted in a significantly higher success rate for LN removal (99%) compared with the conventional approach (69%) [13]. A similar navigation setup was applied during robot-assisted sentinel node biopsies, showing successful identification of 91% of the sentinel nodes using navigation [14]. While this study showed feasibility of navigation in robotic surgery, the workflow for registering the three-dimensional (3D) model using an intraoperative cone-beam CT scan was still cumbersome and time-consuming.

Alternatively, intraoperative ultrasound (US) could be applied for real-time patient registration while minimally interrupting the clinical workflow [15, 16]. In open procedures, registration based on the pelvic bone already proved accurate using either percutaneous or sterile intra-abdominal US. A median target registration error (TRE) of 2.6 (1.3–5.7) mm at pelvic LNs was achieved using this method [16]. Similarly, in a robotic setting, a drop-in US probe could be applied to intra-abdominally image the bone surface for registration. However, clinical implementation is more challenging in robotic surgery compared to open surgery due to the limited field of view and US probe handling using robotic instruments. Therefore, the feasibility and accuracy of this method should be further investigated in robot-assisted surgery.

In this study, we implemented real-time surgical navigation with intraoperative US registration in RA-PLND and RA-RPLND. We evaluated the navigation accuracy at target LNs and usability of the system using surgeon questionnaires. The aim was to evaluate if surgical navigation can improve surgical decisiveness in RA-PLND and RA-RPLND to localize and remove all suspected metastatic LNs.

## Materials and methods

### Study design

We conducted a prospective feasibility study at the Netherlands Cancer Institute in Amsterdam. The study protocol was approved by the institutional medical ethics committee (NL78660.031.21) and registered in ClinicalTrials.gov (NCT05637346). The procedures, including obtaining informed consent, were conducted according to the principles of the Declaration of Helsinki. Patients were recruited between December 2024 and December 2025 and gave written informed consent. Inclusion criteria were patients aged 18 years and older, scheduled for RA-PLND or RA-RPLND, and one or more suspected metastatic LNs on diagnostic (PET-)CT imaging. Exclusion criteria were a cardiac pacemaker, defibrillator or metal implants that influence the CT-imaging quality.

### Navigation setup

**Fig. 1:**
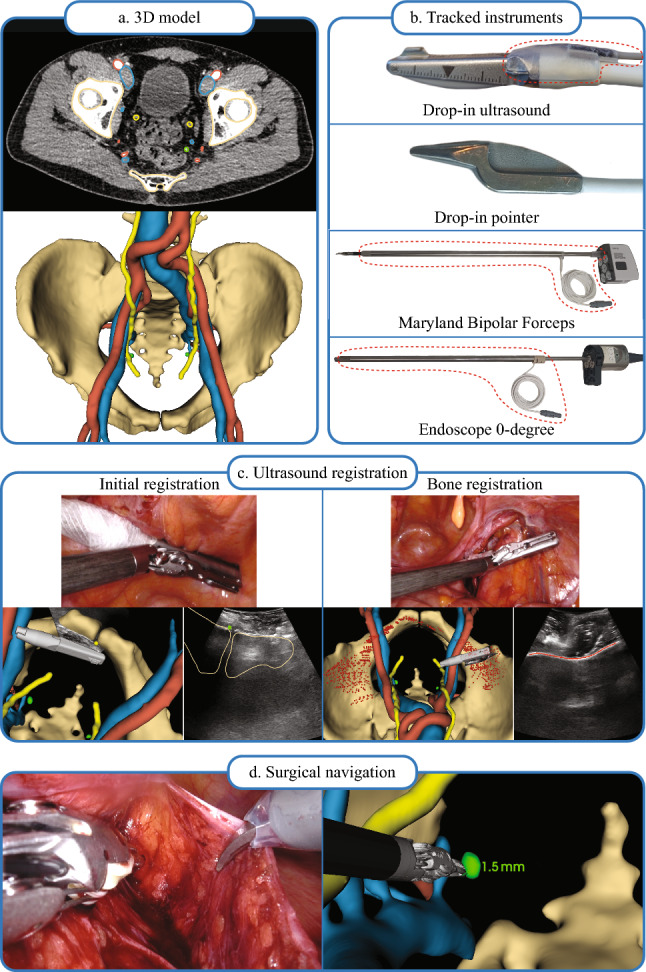
Overview of the navigation workflow. **a** 3D model delineated from a preoperative CT scan with two target lymph nodes in green. **b** Tracked instruments used during surgery (red dashed line highlights the tracker attached to the device). **c** Initial registration using a point selected on the pubic bone in CT (yellow point) and ultrasound (green point) & bone registration using automatic segmentation of the bone surface on ultrasound (red line and points). **d** Navigation view showing the distance of the tracked instrument to the target lymph node (green)

Preoperatively, a patient-specific 3D model was generated from the most recent available diagnostic (PET-)CT scan, which was obtained after any neoadjuvant treatment. The bones, arteries, veins, ureters, and target LNs (Fig. 1a) were semi-automatically segmented by a technical physician using 3D Slicer software [17].

All surgical procedures were performed using the da Vinci® Xi robotic system (Intuitive Surgical, Sunnyvale, CA, USA). The surgical navigation setup consisted of electromagnetic (EM) tracking and intraoperative US. An EM field generator (Planar, NDI Aurora V2) was positioned beneath the operating table, and patient trackers (Philips PercuNav) were used to compensate for patient movement during navigation. Custom-made EM trackers were attached to four surgical tools, including a drop-in US transducer (Rob12C4, BK5000), a drop-in pointer, the da Vinci® endoscope, and one of the da Vinci® instruments, such as the Maryland or fenestrated bipolar forceps (Fig. 1b). This enabled real-time intraoperative tracking of surgical instruments and the camera position.

The navigation software (based on PLUS, SlicerIGT and 3D Slicer [17–19]) integrated the preoperative 3D model, intraoperative US images, and tracked instruments into a single navigation interface. This interface was displayed to the surgeon via the da Vinci® TilePro™. In addition, an artificial intelligence (AI) network (U-Net) was implemented in the software to automatically detect the bone surface from US imaging in real-time. This AI network was trained on a dataset of 56 patients (6573 US images) prior to this study [15, 16]. All navigation data were recorded for offline analysis.

### Study measurements

After docking of the robotic system, the preoperative 3D model was aligned with the intraoperative location of the patient using an automated US-based bone registration method, adapted from our previous work in open surgery [16]. First, the surgeon acquired a US image of an anatomical landmark, either an arterial bifurcation or the pubic bone, which was aligned with the corresponding location in the preoperative 3D model (Fig. 1c). The orientation was automatically determined using the position of the EM field generator. This approach enabled a fast and straightforward initial registration, allowing real-time visualization of the US plane in relation to the 3D model. Second, the patient’s pelvic bones (iliac crest and pubic bone) were scanned using the drop-in US transducer. For patients with retroperitoneal target LNs, the lumbar vertebrae were additionally scanned. The bone surface was automatically detected using the AI network and registered to the preoperative CT bone model, resulting in the final registration of the navigation system (Fig. 1c). The registration accuracy was quantified using the fiducial registration error (FRE), defined as the root mean square distance between the registered US-derived bone surface and the CT bone model. The time required to perform the registration was recorded as well (see Video, Supplemental Digital Content 1, for the US registration workflow).

**Fig. 2:**
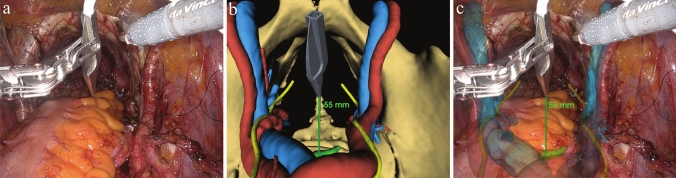
Example of the surgical navigation view in the robotic console. The surgeon sees the original camera image (**a**) together with either the 3D model navigation view (**b**) or the augmented reality navigation view (**c**). Intraoperatively, the navigation view could be adapted based on surgical preferences

After registration, the surgeon verified the alignment by comparing the US images with the overlaid 3D model and by confirming the location of major vascular landmarks using a tracked instrument or drop-in pointer. The tracked instrument or pointer was subsequently used to navigate toward the target LNs, while the navigation system continuously displayed the shortest distance to the selected LN (Fig. 1d). The navigation information could be viewed as an interactive 3D model or as an augmented reality (AR) overlay superimposed on the endoscopic image (Fig. 2). Both views automatically followed the position and orientation of the tracked endoscope and could be switched intraoperatively according to the surgeon’s preference (see Video, Supplemental Digital Content 2, for surgical examples).

The primary aim of the study was the navigation accuracy to localize target LNs, which was defined by the target registration error (TRE). When a target LN was localized, the surgeon pointed at the surface of this LN using the tracked instrument or pointer, and its location was recorded in the software. The TRE was computed as the shortest distance between the recorded location and the surface of the 3D model of the target LN. Negative TRE values, i.e., pointing inside the LN, were considered as 0 mm.

Postoperatively, the surgeons completed a questionnaire about the usability of the navigation system using the system usability scale (SUS), a comparison with the conventional approach on a five-point Likert scale, and general questions about the navigation technique. Three or more points on the Likert scale indicated added value in favor of navigation in comparison to the conventional technique, and a SUS score of 70 or higher indicated acceptance of the navigation technique [20].

### Analysis

LN dimensions were computed from the CT-based 3D models using automated analysis. The maximum and minimum diameters were determined along the longest and shortest axes of the LNs [21], and the LN volume was calculated from the 3D surface geometry [22].

The Shapiro–Wilk test was used to test normality of the data. Normally distributed data were reported as the mean with standard deviation (SD), while non-normally distributed data were reported as the median with interquartile range (IQR). Values of *p* < 0.05 were considered statistically significant.

## Results

The characteristics and surgical outcomes of 20 included patients with 40 target LNs are listed in Table 1. Of all target LNs, 39/40 (98%) were successfully found using navigation and surgically removed. Due to obstructing adipose tissue in a patient with a BMI of 52 kg/m^2^, one para-aortal LN was surgically inaccessible, unrelated to the navigation system. While three postoperative Clavien–Dindo grade II complications were observed, including inguinal wound infection, urosepsis, and upper-extremity thrombophlebitis, none were related to the navigation system.

**Table 1:** Patient characteristics, lymph node characteristics and surgical outcomes after robot-assisted pelvic and retroperitoneal lymph node dissection with surgical navigation

Characteristic	Value
*Patient characteristics (n* = *20)*	
Age at surgery (years), mean ± SD	64.4 ± 10.5
BMI (kg/m^2^), median (IQR)	24.5 (23.0–30.6)
ASA score, median (IQR)	2 (2–2)
Previous abdominal surgery, n (%)	5 (25%)
Gender, n (%)	
Male	17 (85%)
Female	3 (15%)
Patient position, n (%)	
Trendelenburg	16 (80%)
45° lateral decubitus	4 (20%)
Primary tumor, n (%)	
Skin cancer	8 (40%)
Testicular cancer	3 (15%)
Prostate cancer	2 (10%)
Penile cancer	2 (10%)
Bladder cancer	2 (10%)
Rectal cancer	1 (5%)
Renal cancer	1 (5%)
Endometrial cancer	1 (5%)
Number of target LNs per patient, n (%)	
1	11 (55%)
2	4 (20%)
3 or more	5 (25%)
*Lymph node characteristics (n* = *40)*	
LN volume (cm^3^), median (IQR)	1.0 (0.4–2.3)
Minimum LN diameter (cm), median (IQR)	0.9 (0.7–1.2)
Maximum LN diameter (cm), median (IQR)	2.3 (1.3–3.0)
Target LN location, n (%)	
External iliac	18 (45%)
Obturator	8 (20%)
Aortic	6 (15%)
Caval	2 (5%)
Pararectal	2 (5%)
Presacral	2 (5%)
Common iliac	2 (5%)
*Surgical outcomes (n* = *20)*	
Operative time (min), median (IQR)	185 (163–269)
Estimated blood loss (ml), median (IQR) (*n* = 16)	35 (1–50)
Intraoperative complications, n (%)	0 (0%)
Postoperative complications, n (%)	
Clavien–Dindo < III	3 (15%)
Clavien–Dindo ≥ III	0 (0%)
Pathological nodal metastasis, n (%)	13 (65%)

*ASA* American Society of Anesthesiologists; *BMI* body mass index; *IQR* interquartile range; *LN* lymph node; *SD* standard deviation

### Registration time

The total time spent on US bone registration was 7 (5–10) min, of which 2 (1–2) min were required for the initial registration. In four patients, lumbar vertebrae were included for registration because of retroperitoneal target LNs. The registration times of those cases were 8, 9, 11, and 11 min.

### Navigation accuracy

**Fig. 3:**
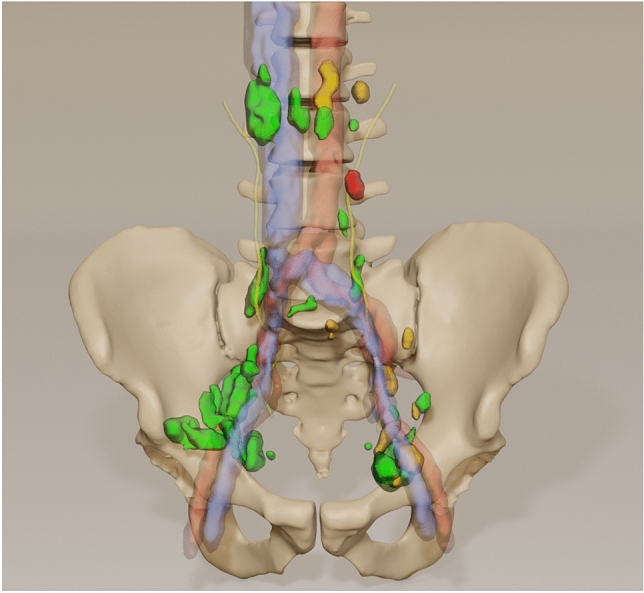
Compilation of the 40 target lymph nodes relative to a 3D model and their localization accuracy (target registration error) according to the navigation system (green: ≤ 3 mm, orange: > 3 and < 8 mm, red: surgically inaccessible)

The US registration resulted in a mean FRE of 1.0 ± 0.2 mm at the bone surface. At the 39 localized target LNs, the median TRE was 0.8 (0.0–3.0) mm. Figure 3 shows the location of these LNs within the patient’s body, including the inaccessible para-aortal LN (see Video, Supplemental Digital Content 3). No correlation was found between the TRE and the FRE, BMI or LN volume and diameter.

### Surgeon satisfaction and system usability

**Fig. 4:**
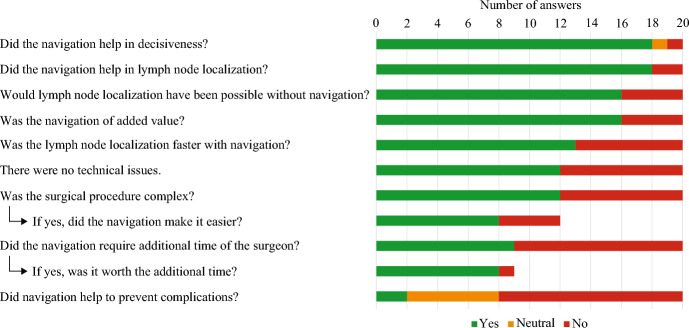
Results of the general questions of 20 questionnaires answered by nine different surgeons regarding the use of navigation during robot-assisted pelvic and retroperitoneal lymph node dissection

Twenty targeted robot-assisted lymphadenectomies were performed by nine surgeons representing four specialties (urology, *n* = 10; surgical oncology/melanoma, *n* = 8; gynecology, *n* = 1; colorectal surgery, *n* = 1), and all 20 questionnaires were completed. Answers to the general questions regarding surgical navigation are summarized in Fig. 4. Although surgeons reported that LN localization would have been possible without navigation in 80% (16/20) of the procedures, navigation improved decisiveness and LN localization in 90% (18/20) of the procedures. Surgeons reported that intraoperative navigation facilitated efficient localization of LNs, assessment of anatomical relationships with adjacent structures, such as vessels, and identification of the LN dissection template margins.

Twelve procedures (60%) were considered complex, of which navigation simplified 67% (8/12). Additional time for navigation was reported in 9 procedures, but was considered worthwhile in 89% (8/9). Surgeons reported technical issues in 8 procedures, involving software (2), connection errors (3), and tracked instruments (3). However, none of these issues resulted in complications and they only caused a slight delay before the surgeon was able to proceed using the navigation system.

The surgeon’s experience using the navigation technique was more positive compared to the conventional approach with a mean Likert score of 3.4 ± 0.5. Especially certainty in decision-making (4.0 ± 0.6) and localization of LNs (3.9 ± 0.7) scored high (see Figure, Supplemental Digital Content 4, for all sub-questions). Regarding total operating time, the surgeon’s experience was slightly more negative compared to conventional (2.6 ± 0.9).

The median SUS score was 70 (64–73), indicating above average acceptance of the navigation setup (see Figure, Supplemental Digital Content 5, for all sub-questions). Surgeons indicated that they would like to use the system regularly (4.2 ± 0.5 on a scale of 1–5), but support from technical staff is still required (3.7 ± 0.7 on a scale of 1–5).

## Discussion

This study proves that surgical navigation is feasible in RA-PLND and RA-RPLND with a high accuracy of 0.8 (0.0–3.0) mm at target LNs. We showed that the technique can safely be applied in different oncological fields in the abdominal-pelvic area, such as urology, gynecology, and general surgery, to improve surgical decisiveness and localization of the LNs. This ensures that the target (PET positive/CT enlarged) LNs are localized and removed with certainty, potentially improving the patient’s survival. An example application can be found in prostate cancer for the resection of prostate-specific membrane antigen (PSMA) PET positive LNs, which are often smaller LNs containing micro-metastasis [23, 24]. This was highlighted in one of the patients included in this study who had two small PSMA-PET positive pararectal LNs (Fig. 1a). Despite the use of a drop-in gamma probe, the surgeon stated that he could not have found both LNs without navigation. Pathological analysis confirmed metastatic prostate cancer in the resected PSMA-PET positive nodes, highlighting the additional clinical value of navigation.

Although surgeons commonly perform robot-assisted lymphadenectomy according to a standardized anatomical template, e.g., limited, standard, or (super-)extended pelvic LN dissection [25], surgical navigation could still provide added value. By integrating a standardized LN template map within the 3D model, navigation can display anatomically defined nodal stations intraoperatively, supporting structured resection for reproducible cancer staging [26]. In particular, navigation can assist in accurately identifying the most cranial and caudal LNs, thereby supporting precise determination of the template dissection boundaries. Furthermore, navigation enables confirmation that a preoperatively identified (malignant) target LN is encompassed within the template resection margins, improving surgical decisiveness and ensuring completeness of resection. On the other hand, new approaches suggest that therapeutic LN dissection may be safely omitted in patients with a major pathological response, with the aim of reducing morbidity and improving quality of life [27, 28]. In melanoma, for example, there is an increasing shift toward selective removal of an index LN after neoadjuvant therapy without therapeutic LN dissection. In this setting, surgical navigation may facilitate precise intraoperative localization of this index LN, adding clinical value for individualized and response-adapted surgical strategies in the future.

Limited literature is available on using tracking systems in robotic lower abdominopelvic surgery [29]. Besides the feasibility study of our group in robot-assisted sentinel node biopsy [14], only one other study was found in which a comparable navigation technique was used. They applied an optical tracking system for lateral RA-PLND in five patients [30]. While they showed feasibility of tracking the camera and registering the 3D model using manual intra-abdominal landmark selection, their TRE (10.0–12.6 mm) was more than 10 times higher than our median TRE using US bone registration (0.8 mm). This could be explained by the fact that they used arterial bifurcations for landmark registration, which is more susceptible for deformation compared to the bone surface. We also showed this in an earlier study comparing different registration methods in open abdominopelvic surgery: a TRE of 8.0 mm after arterial bifurcation registration compared to a TRE of 2.6 mm after bone registration [16]. Therefore, registration based on rigid structures, such as bone, is recommended.

More literature is available on image-guided surgery without EM tracking, such as virtual reality, mixed reality, and AR, especially in the urological field [31]. However, many studies focus on preoperative planning and surgical training using 3D models (in virtual reality) [32–34]. Regarding AR, one study described a novel AR-PSMA-3D technique for intraoperative identification of PSMA-PET positive LNs in 13 patients [35]. While their AR visualization is similar to our approach, they do not track the location of any instrument and require manual registration of the 3D model onto the surgical camera view, which is a time-consuming process susceptible to human errors. Therefore, we believe that combining AR with instrument tracking and automatic registration is necessary for an accurate and robust navigation system. This would enable broader applications of surgical navigation beyond lymph node localization, including tumor margin assessment in procedures such as robot-assisted partial nephrectomy, radical prostatectomy, and hepatic surgery.

For TRE assessment, manually pointing at target LNs with a tracked pointer introduced some limitations. LNs were sometimes partially covered by adipose tissue, potentially leading to underestimation of the true TRE, as the pointer tip could not always be placed directly on the LN boundary. Furthermore, TRE was measured at only a single point, providing no information about the accuracy of the full three-dimensional shape or potential local registration errors. Nevertheless, this point-based evaluation aligned with the primary goal of this study of accurate LN localization rather than precise shape correspondence. From a surgical perspective, correct spatial LN localization is sufficient to guide dissection and decision-making, making the chosen TRE assessment clinically relevant.

In the questionnaires, surgeons reported a modest increase in operative time using navigation compared to conventional procedures. On the other hand, they indicated that the additional time was generally worthwhile and helps in LN localization and surgical decision-making, which reduces the operating time. Therefore, the time gained with faster LN localization and decision-making could compensate for the time required for setting up the navigation.

The use of wired tracked instruments could be cumbersome during robot-assisted procedures, potentially restricting instrument handling and interfering with the surgical workflow. In the future, integrated tracking into the robotic system or wireless tracking could overcome these challenges. While most wireless systems are still in development [36, 37], Elucent Medical recently released a wireless navigation system EnVisio™ for intraoperative localization of nonpalpable breast and axillary lesions, which has potential for implementation in robot-assisted surgery [38]. Alternatively, simultaneous localization and mapping (SLAM) techniques should be explored. SLAM enables instrument tracking and anatomical reconstruction directly from endoscopic images, potentially eliminating the need for external tracking systems and supporting real-time navigation. Although early studies show promising accuracy of this technique, further research is needed to address technical challenges and validate clinical applicability [39, 40].

An important consideration for broader clinical implementation of this navigation setup is the requirement for specialized equipment, including an EM tracking system with customized adapters for the robotic instruments. While these components were of added value for navigation in the current research setting, the workflow could be simplified to facilitate broader adoption. For example, navigation based solely on a tracked pointer could reduce hardware requirements and setup complexity, although at the expense of advanced functionalities such as instrument tracking and AR visualization. Additionally, the AI model used for automatic US bone segmentation could be easily copied and trained on new data. Since bone surfaces are readily identifiable on ultrasound, a relatively small dataset would be sufficient for this application. Finally, the current workflow required the presence of a technical physician to set up and supervise the navigation system. Although this is common during the clinical evaluation of non-commercial technologies, increased automation and integration into commercial robotic platforms are expected to reduce the need for dedicated technical support in the future.

In conclusion, EM-tracked navigation enabled highly accurate localization of pelvic and retroperitoneal LNs during robot-assisted lymphadenectomy, which resulted in successful integration of this technique into standard-of-care RA-PLND and RA-RPLND procedures at our institute. With ongoing technological advancements, this approach holds strong potential for broader clinical adoption of image-guided navigation.

## Supplementary Information

Below is the link to the electronic supplementary material.

Supplementary file1 (MP4 7748 KB)

Supplementary file2 (MP4 8467 KB)

Supplementary file3 (MP4 3754 KB)

Supplementary file4 (PDF 243 KB)

Supplementary file5 (PDF 310 KB)
