# Supplementary material for: Real-time surgical navigation for intraoperative lymph node localization during targeted robot-assisted pelvic and retroperitoneal lymph node dissection
Source: Surg Endosc. 2026 Jul 27;40(8):7223–31. doi: 10.1007/s00464-026-13187-5 (PMC13457233; doi:10.1007/s00464-026-13187-5)
Supplement: Supplementary file 1 [file 464_2026_13187_MOESM4_ESM.pdf]

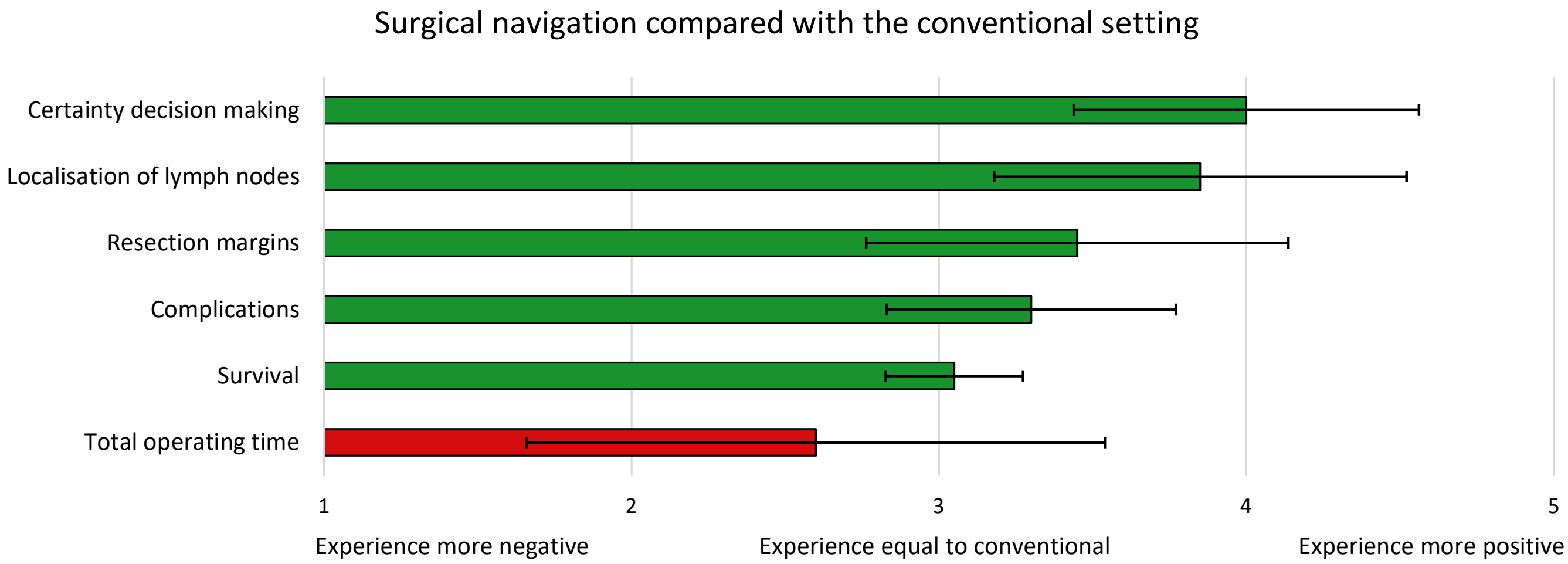

Supplemental Digital Content 4: Surgeon’s experience of the navigation technique compared with the conventional setting on a 5-point Likert scale. Mean scores with standard deviation are shown, based on 20 questionnaires completed by nine surgeons.
